# Awareness of age-related change and its relationship with inductive reasoning and ageism

**DOI:** 10.1093/geroni/igab046.2309

**Published:** 2021-12-17

**Authors:** Abigail Voelkner, Grace Caskie

**Affiliations:** 1 Lehigh University, Easton, Pennsylvania, United States; 2 Lehigh University, Bethlehem, Pennsylvania, United States

## Abstract

Subjective aging is important due to its relationship with well-being. Diehl and Wahl (2010) proposed Awareness of Age-Related Change (AARC) as a measure of subjective aging; their theoretical model proposed that cognition’s relationship to AARC is mediated by ageist experiences. The current study tests this model and proposes an alternative model where cognition is hypothesized to mediate the relationship of ageist experiences to AARC. Inductive reasoning was used to measure cognition due to its susceptibility to ageism. Participants were 283 older adults aged 66-90 years (M=69.08, SD=3.36) without a dementia diagnosis or cognitive impairment. Inductive reasoning was measured by Word Series, Number Series, Letter Sets, and a composite score. AARC total losses, cognitive losses, total gains, and cognitive gains were used. Age, gender, and education covariates were included. Analysis of Diehl and Wahl’s (2010) model showed that the composite and individual reasoning measures had negative direct effects on all AARC measures. Ageism mediated the effect of the composite and individual reasoning measures on AARC total and cognitive losses. In the alternative model, ageist experiences had positive direct effects on AARC total and cognitive losses. The composite, Number Series, and Letter Sets mediated the effect of ageism on all AARC measures. Word Series mediated the effect of ageism on total and cognitive losses. Overall, inductive reasoning seems to play an important role in understanding the relationship of ageism with AARC. Thus, inductive reasoning abilities may be a potential intervention point to cultivate well-being. Future research should assess additional domains of cognition.

